# Editorial: Biological aspects of targeted drug discovery: Development of novel targets and/or chemotherapies, and drug repurposing

**DOI:** 10.3389/fonc.2022.1106610

**Published:** 2022-12-22

**Authors:** Rajkumar Singh Kalra, Sandeep Singh, Anjana Munshi, Jitender Bariwal

**Affiliations:** ^1^ Okinawa Institute of Science and Technology, Graduate University, Okinawa, Japan; ^2^ Central University of Punjab, VPO Ghudda, Bathinda, Punjab, India; ^3^ The University of Texas MD Anderson Cancer Center, Houston, TX, United States; ^4^ University of Nebraska Medical Center, Omaha, NE, United States

**Keywords:** cancer, drug discovery, chemotherapy, targeted medicine, drug repurposing, bioactive compounds, natural products, cancer therapeutics

Cancer drug discovery is a competitive research area in interventional cancer research. It comprises elements of biology and chemistry for developing novel drugs/small molecules demonstrating potent anti-cancer efficacy (1). Enormous efforts across laboratories and pharmaceutical industries worldwide identify thousands of such molecules annually, however, only a few reach the clinical testing stage (2). It reflects a high failure rate for several reasons that include (yet may not be limited to) drug efficacy, physiological effects, associated side effects, and drug toxicity to normal cells (3). Targeted drug discovery approaches advanced the development of refined drugs/molecules with high therapeutic potential, yet again the success rate remains limited (4). Investigation into the drugs that are approved to cure certain diseases/conditions at times were unexpectedly seen to provide benefits from unintended side effects (5). Availability of the safety, pharmacokinetic, and manufacturing data of licensed drugs/molecules enables researchers to extract/concentrate and investigate their ‘unintended’ beneficial effects, avoiding a longer procedure of preclinical and clinical trials for safety evaluation (6, 7). Above practice in drug designing and discovery is generally called ‘drug repurposing’ or ‘drug repositioning’ (8). Given the availability of relevant safety data that skips time and funding, drug repurposing offers opportunities for researchers to evaluate their anti-cancer and preventive efficacies against routine chemotherapeutic drugs/therapies (9). In the present Research Topic, we gathered new knowledge on novel anticancer compounds/molecules, research strategies/approaches, and repurposing drugs.

Given the safety and accessibility, natural bioactive compounds/extracts from herbal medicinal plants attained larger interest in the field of cancer drug discovery. In an original report, Li et al. explored the anti-cancer potential of Shengxian Decoction (SXT, a traditional Chinese medicine) against lung adenocarcinoma. Extraction and analysis of crude SXT led them to reveal the abundance of mangiferin, i.e., an established anti-cancer compound. The serum pharmacological analysis exhibited growth-suppressive activity of serum SXT against A549 lung cancer cells that was further validated in the animal model. At the molecular level, their investigation revealed tumor necrosis-inducing and HIF-1α suppressive function of SXT. Their investigation provided the first scientific evidence of the anti-cancer efficacy of SXT against lung adenocarcinoma. Another report by Lai et al. identified the anti-leukemia activity of honokiol (a natural small biphenolic phytochemical compound) on a panel of acute myeloid leukemia (AML) cell lines. To analyze honokiol’s potent growth arrest (G0/G1) and anti-viability/proliferative activities at the molecular level, they discovered a noncanonical ferroptosis pathway-inducing function of honokiol that upregulates the intracellular lipid peroxide and HMOX1 levels in AML cells. This report suggested that honokiol could serve as a potential ferroptosis activator in AML. In another report, Zuppone et al. genetically modified the plant-derived single-chain ribosome-inactivating protein saporin (SAP) structure by joining its N-terminus to the ACDCRGDCFCG peptide (RGD-4C, an αv-integrin ligand), and subsequently analyzed the anti-tumor efficacy of the resulting protein *viz.* RGD-SAP *in vitro* and *in vivo* mouse model. They revealed that RGD-4C targeting domain fusion to SAP enhances the cytotoxic activity of the latter in an αv-integrin expression-dependent manner. Of note, they showed that a systemic administration of RGD-SAP with mitomycin C (a frontline chemotherapeutic drug to treat bladder cancer) extended the survival of mice with orthotopic bladder cancer, lacking any systemic toxicity. They stressed on exploiting the potent efficacy of RGD-SAP either alone or in combination with the current chemotherapy regimes to treat bladder cancer and potentially other solid tumors. In this line, another study by Liang et al. using network pharmacology and wet lab attempted to identify the potential therapeutic targets of Asparagus (ASP) in colorectal cancer (CRC). They identified 9 active components from ASP, while its 157 potential targets were predicted including the p53, FOS, AP-1, and Akt1. The obtained results from *in vitro* and *in vivo* assays affirmed the anti-cancer activities of ASP, thereby providing the first evidence of its potential role for CRC therapeutics. Consistent with the emerging significance of natural bioactive compounds in the complementary or alternative therapeutics, Mitra et al. in a review report further assessed the anti-cancer potential of Diallyl disulfide (DADS), a key bioactive organosulfur compound from garlic. Garlic is widely regarded for its medicinal properties that have a wide range of bioactivities including antithrombotic, hypo-lipidemic, antibacterial, and anticancer, while DADS exhibited prominent anti-tumor activities against diverse cancers. The gathered information comprehensively provided insights into the anti-cancer properties of DADS, its mode of action, mechanisms, bioavailability, and pharmacokinetics from existing studies. To this end, Rahman et al. in a detailed review further explored the recent trends in natural products-led perturbed cellular signaling of a cancer cell. The report reviewed the role of natural compounds in causing cell cycle arrest, triggering intrinsic and/or extrinsic apoptosis pathways. The report highlighted the significance of several key signaling pathways and their effector proteins including MAPK, NF-B, Wnt, Akt, Notch, ER, and p53, which regulate apoptotic signals specifically in pre-malignant or cancer cells. They underlined the importance of non-toxic “natural drugs” for cancer prevention and therapeutics. Taken together, original research and reviews in the section enhanced our understanding of the anti-cancer activities of natural compounds at the cellular and molecular levels.

Emergence of targeted drug discovery approaches highlighted the significance of selective inhibitors, small molecules/metabolites, and novel peptides in cancer therapeutics. In this line, original research from Yu et al. elucidated the potent function of Ag120 (ivosidenib, a mutant isocitrate dehydrogenase 1 or IDH1 inhibitor) as an ASCT2 (solute carrier family 1 member 5) inhibitor in colorectal cancer. In the cancer cell metabolic reprogramming, glutamine metabolism plays an important role in tumor progression, which underlines that targeting glutamine uptake (via the transporter protein ASCT2/SLC1A5) could be an effective interventional strategy. The report showed that Ag120 blocks glutamine uptake, metabolism and restricts tumor cell growth *via* ERK and mTOR signaling pathways. These efforts identified a novel mechanism of Ag120 function as an ASCT2 inhibitor for cancer therapeutics. In another original report, Olaechea et al. studied the associations between prior long-term use of NSAIDs (non-steroidal anti-inflammatory drugs) or glucocorticoids with cachexia (i.e., an inflammatory and metabolic syndrome of unintended weight loss in muscle and adipose tissue) incidence and occurrence of post-diagnosis weight loss in a retrospective cohort of 3,180 cancer patients. Of note, they elucidated that the prior anti-inflammatory treatments, mainly NSAIDs, have protective effects against the manifestations of cachexia at the cancer diagnosis. They stressed on the requisite of detailed investigation to precisely assess the potential therapeutic benefits of NSAIDs early in cancer management. Examining the repurposing of Metformin (a first-line drug for type 2 diabetes), a report by Fang et al. further investigated inhibitory effect on the medulloblastoma (MB) and elucidated its efficacy in the Sonic hedgehog (Shh) subgroup MB cell line *in vitro* and *in vivo*. Of note, these effects were shown to cause by AMPK-mediated inhibition of the Shh signaling pathway. In the case of hemangioblastoma (HB, a highly vascularized cystic tumor of the CNS or central nervous system), a case report by Jin et al. revealed that anlotinib, i.e., a tyrosine kinase inhibitor (targeting VEGFR) can produce the significant radiographic response. The 62-year-old woman patient with multiple recurrent lumbar and sacral cord HBs post receiving the 3 months anlotinib treatment showed marked tumor regression, hinting anlotinib could be a therapeutic approach for patients with multiple recurrent HB or multiple lesions/VHL disease. Amid increasing drug repurposing in clinical practice, a careful review of drug safety for cancer patients is important. In a perspective report, Embaby et al. underscored the need for diligence when choosing the β-adrenergic receptor blockers (β-blockers) in angiosarcoma. The β-blockers/antagonists are often prescribed in ischemic cardiac disease due to their anti-hypertensive and anti-arrhythmic activities. Given the usage of β-blockers in recent times to improve therapeutic options for angiosarcoma patients, it is crucial to determine the optimal pharmacological activities of β-blockers in the patient cohort where most patients lack cardiovascular co-morbidity. The report underlined the need to use β-blockers that have intrinsic sympathomimetic or vasodilator effects, e.g., labetalol, pindolol or carvedilol, essentially to minimize the risk of adverse cardiovascular events. To ensure efficient drug delivery, original research by Mishra et al. developed water-dispersible and biocompatible nanocargo (GO-PEG) for Glioblastoma multiforme (GBM; a primary malignant form of CNS tumor in adults). Given the utility of nanocarriers that improve a drug’s water dispersibility, cellular permeability, and efficacy at a lower dose, they developed a GO-PEG nanocarrier based on modified graphene oxide with a poly(ethylene glycol) amine dendrimer (6-armed) for effectively load and carry two hydrophobic anticancer drug molecules *viz.* CPI444 and vatalanib targeting A2AR, VEGFR, PDGFR, and c-KIT. They validated the above regime to reduce cancer cell viability, migration/invasion, calcium levels, and expression of markers (Oct4 and Nanog) associated with GBM, and thereby affirmed the utility of the adopted strategy toward effective GBM treatment. The revised/repurposed targeting approaches against cancer are of much interest, yet tumor resistance has been an evident challenge. In this line, original research from Liu et al. attempted to overcome FXYD3 (an FXYD protein linked with Na+/K+-ATPase α/β heterodimers that protect β1 subunit against glutathionylation that destabilizes the heterodimer and inhibits its function) -led treatment resistance in cancer cells. They developed two FXYD3 peptide derivatives *viz.* FXYD3 -pep CKCK and -pep SKSK that allow elimination and increase of FXYD protein function, respectively. The report revealed that Cys residue is critical to counter β1 subunit glutathionylation to promote the cytotoxicity upon siRNA-mediated FXYD3 suppression. Chen et al. in a review report, further assessed the functional significance of membrane-associated E3 ligases in cancer and as a drug target. Authors shed light on the critical function of the ubiquitin E3 ligase in facilitating intracellular communication, while linking its dysfunction to cancer. Therefore, E3 ligase could as a novel drug target for cancer therapy, where drugs/small molecules inducing ubiquitination-mediated degradation of the cancer-cell membrane may be of prime interest. To enable the virtual and reliable identification of targets for drug discovery, Wei et al. in a method article further developed a graphical web interface *viz.* TAIGET, which comprises a docking, a target screen, and an annotation module respectively. TAIGET consist of a robust target annotation module (curating >14,000 cancer-related works, having 73 cancer types and 2,170 cell types), and is freely accessible at http://www.taiget.cn/. Also, it has a comprehensive interface to support researchers those lack technical expertise in the virtual drug discovery practice.

Taken together, the present Research Topic collects knowledge from the submitted original research article(s), reviews, case reports, method and perspective reports and comprehensively presents new findings on the anti-cancer drug (natural compounds, inhibitors/peptides, and small molecules) discovery, novel regimes, and emerging clinical significance of drug repurposing.

## Author contributions

All authors made direct and intellectual contributions to the report and provided their approval for the publication.
